# Drug utilisation of antipsychotics and lithium in Sweden 2008–2021 – a nationwide study of children aged 5–17 years

**DOI:** 10.1007/s00787-026-02960-5

**Published:** 2026-01-10

**Authors:** Elin Dahlén, Håkan Jarbin, Anders Sundström, Ida Walles, Sverre Wikström, Elin Eyfells Kimland

**Affiliations:** 1 Swedish Medical Products Agency, Uppsala, Sweden; 2 Department of Pharmacy, Faculty of Pharmacy, Uppsala University, Uppsala, Sweden; 3 Unit for Child and Adolescent Psychiatry, Department of Clinical Sciences Lund, Lund University, Lund, Sweden; 4 Department of Child and Adolescent Psychiatry, Region Halland, Halmstad, Sweden; 5 Uppsala University Hospital, Uppsala, Sweden; 6 Centre for Clinical Research, Region Värmland, Sweden; 7 School of Medical Sciences, Örebro University, Örebro, Sweden

**Keywords:** Child, Antipsychotics, Drug utilisation, Nationwide study, Register data

## Abstract

Antipsychotics are mainly used for severe psychiatric disorders. Risperidone is the only antipsychotic approved for younger children. Aripiprazole is approved for older children, ziprasidone and lurasidone for adolescents. To describe paediatric utilisation of antipsychotic drugs in Sweden. Dispensations of antipsychotic drugs to children aged 5–17 years from 2008 to 2021 were analysed using the National Prescribed Drug Register and recorded diagnoses were obtained from the National Patient Register. Overall, dispensations of antipsychotics to children increased from 16.7 to 36.9/10,000 between 2008 and 2021. The increase was larger in girls, from 12.3 to 36.6/10,000. New users of antipsychotics increased by 121% in the same period, from 7.9 to 17.5/10.000. In 2019, in children aged 12–17, 63% of girls and 71% of boys were dispensed had at least three dispensations of antipsychotics, while the corresponding proportion in children aged 5–11 were 52% for girls and 63% for boys. Risperidone was the most common drug in boys of all ages and in girls aged 5–11. In girls aged 12–17, aripiprazole and quetiapine were most common. The proportion of on-label antipsychotic use was 21% in girls and 15% in boys based in authorised product information and increased to 37% and 45%, respectively, when aligning with national guidelines. The use of antipsychotics has increased, with the proportion of girls starting treatment at the end of study period exceeding that of boys. A considerable proportion of prescriptions was off-label, even when considering national guidelines.

**Supplementary Information:**

The online version contains supplementary material available at 10.1007/s00787-026-02960-5.

## Introduction

Antipsychotic drugs and lithium are of importance and widely used for treatment of a broad range of behavioural and mental health disorders in children. Most studies regarding the effect and safety of antipsychotic drugs focus on schizophrenia and mania, which are uncommon in children [1–3]. The number of children with a psychiatric diagnosis has increased, primarily for attention-deficit hyperactivity disorder (ADHD), depression, and anxiety disorders [2]. The number of children with a diagnosis of schizophrenia or some other non-mood psychotic disorder has increased, although it remains small [4]. A few antipsychotics are approved by the European Medicines Agency (EMA) for use in children and adolescents, Supplementary Table S1. Risperidone is approved for short-term treatment of conduct disorders from the age of 5 years in children with intellectual disability. Ziprasidone is approved for treating manic episodes in bipolar disorder in children from age 10. Aripiprazole is approved for treating manic episodes in bipolar disorder in children from age 13 years and schizophrenia from age 15. Lurasidone is approved for treating schizophrenia in children from age 13 [5].

Adverse drug reactions of antipsychotics are common and sometimes severe [3]. Children are at greater risk than adults of experiencing severe side effects from antipsychotics, particularly metabolic effects, including, significant weight gain, insulin resistance, type 2 diabetes, and hyperlipidaemia [6]. Metabolic side effects can occur already at low doses and rapidly after start of treatment [7].

Antipsychotics have the lowest utilisation rate among prescribed psychotropic drugs in children [8]. A high proportion of off-label prescriptions of antipsychotics to children has been reported; ADHD and conduct disorders were the most common diagnoses [9]. In the Nordic countries, the rate of antipsychotic drug use among children varied between 2.8/1,000 and 9/1,000 during 2004–2018, with a slightly higher rate of antipsychotics among boys than girls [8]. Although children and adolescents are less frequently prescribed antipsychotics than adults, several studies have showed that the prescription of antipsychotics for children has increased markedly in the last 20 years in the Nordic countries [8, 10–13] as well as world-wide [3, 10, 14].

There is a lack of knowledge on trends in antipsychotic use among children especially in relation to indications and off-label use.

### Aims of the study

In this nationwide study, we aimed to describe the drug utilisation patterns of antipsychotics among Swedish children aged 5–17 years, including reported diagnoses, dispensation of other psychotropics and on-label classification.

## Methods

### Study population

The study population comprised all children aged 5–17 years residing in Sweden between 2008 and 2021 with at least one dispensation of an antipsychotic drug. Children aged 0–4 years was excluded due to the small sample size.

### National registers

Data were retrieved by linking of national health data registers using the personal identity numbers assigned to all Swedish residents. The national registers used were the National Prescribed Drug Register [15, 16] and the National Patient Register [17], both held by the National Board of Health and Welfare.

The National Prescribed Drug Register contains data on all prescribed drugs dispensed at pharmacies in Sweden, classified in accordance with the Anatomic Therapeutic Chemical Classification (ATC) system [18]. Drugs administered in hospital care are not included. The National Patient Register (PAR) contains information on diagnoses from hospitalisations and outpatient visits in specialised care. The registered diagnoses are classified in accordance with the Swedish version of the International Statistical Classification of Diseases and Related Health Problems - Tenth Revision (ICD-10-SE) [19].

### Analysis of dispensed drug prescriptions

The analysis included all drugs classified under ATC code N05A (antipsychotics) and categorised as second-generation antipsychotics (SGAs), first-generation antipsychotics (FGAs), or lithium. Child’s age was based on age during the year of dispensation of antipsychotics.

Prevalent users of dispensed antipsychotics were calculated as the total number of children dispensed at least one antipsychotic drug during each calendar year per 10,000 children in the population that year. Individuals were considered new users if at least one antipsychotic drug was dispensed during a calendar year, with no dispensations of antipsychotics in the previous two calendar years. The data for all prevalent and new users were standardised for age and sex in 2021. Additional analyses regarding number of dispensations, psychiatric diagnoses, dispensations of other psychotropics, and on-label use were performed on data from 2019. This year was selected to study the characteristics before the start of the COVID-19 pandemic.

Number of dispensations was classified as one, two or three or more dispensations of an antipsychotic drug during 2019. One dispensation usually covers medication for three months. Thus, persistent users are typically dispensed medications three or four times per year. The antipsychotic prescriptions to children using dose-dispensing services (3% of the study population) were excluded when calculating number of dispensations during a calendar year, because of more frequent distribution normally corresponding to 14 days of use. The number of dispensations was omitted for olanzapine and quetiapine in children aged 5–11 years due to the small numbers of individuals.

All prescription texts related to tablets of the lowest strengths of risperidone (0.25 mg), quetiapine (25 mg), olanzapine (2.5 mg), or aripiprazole (5 mg) were reviewed for children who only filled prescriptions of these lowest strengths during 2019. Texts that explicitly prescribed only one tablet per day were identified. Close to 4,000 prescription texts were reviewed for nearly 2,400 children.

Psychiatric diagnoses were classified in accordance with chapter F `Mental and behavioural disorders` in the ICD-10-SE, down to the 3-character level (e.g., F84 `Autistic disorder`). All psychiatric diagnoses, documented up to two years prior to dispensing at least one antipsychotic drug prescription during 2019, among children aged 5–17 years of age were retrieved. Each diagnosis was counted only once, but each child could have several diagnoses. The categorisation of diagnoses is presented in Supplementary Table S2.

On-label prescription was classified based on both the paediatric labelling in the Summary of Product Characteristics (SmPC) and the available paediatric national guidelines (Supplementary Table S3). On-label prescription analyses were performed for the four most commonly dispensed antipsychotics. A drug was regarded as on-label if it was prescribed within the approved age limit and/or indication described in the labelling in the SmPC authorised by the EMA, using the registered diagnoses in PAR as a surrogate for indication [5]. Dosing information was not included in the definition due to a lack of structured data. For practical reasons, the on-label classifications were based on substance instead of the specific dispensed drug. Therefore, it was necessary to assume that the product that was dispensed represented the widest on-label age interval for that substance. On-label indications according to national guidelines were defined as indications made in accordance with national recommendations published by the Swedish Medical Products Agency [20, 21] or national guidelines from the Swedish Society for Child and Adolescent Psychiatry [22].

Other psychotropic drug use was defined as at least one dispensation during the same calendar year as the antipsychotics, of antidepressants (ATC code N06A), antiepileptics (ATC code N03A), anxiolytics (ATC code-N05B) and phenothiazine derivatives (ATC code R06AD), hypnotics and sedatives (ATC code N05C) or psychostimulants (ATC code N06B).

Data processing and statistical analysis was performed in SAS software, version 9.4.

## Results

**Fig. 1:**
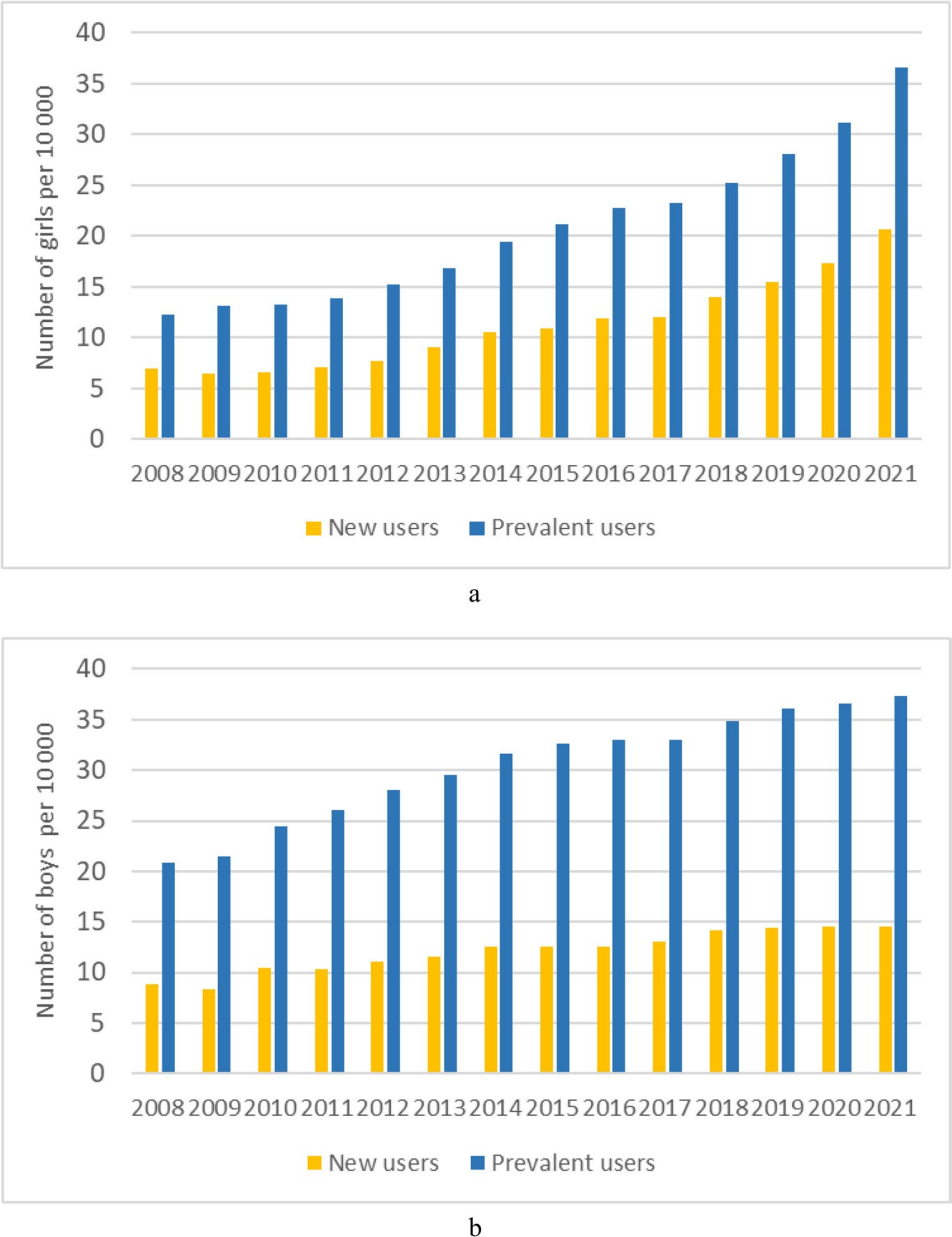
(**a**) Number of prevalent users and new users of antipsychotic drugs among **girls** per calendar year 2008-2021. A prevalent user was defined as having at least one dispensation of antipsychotics by 10 000 children in the population that calendar year. A new user was defined as having a first dispensation of antipsychotic drugs after a drug-free period of at least two years by 10 000 children in the population that calendar year. (**b**) Number of prevalent users and new users of antipsychotic drugs among **boys** per calendar year 2008-2021. A prevalent user was defined as having at least one dispensation of antipsychotics by 10 000 children in the population that calendar year. A new user was defined as having a first dispensation of antipsychotic drugs after a drug-free period of at least two years by 10 000 children in the population that calendar year

On average 1.5 million children aged 5–17 years were resident in Sweden during the study period. We retrieved data on 24,581 children (53% boys and 47% girls) aged 5–17 years who were dispensed at least one antipsychotic drug between 2008 and 2021. The annual numbers of new users and prevalent users of antipsychotics (also presented as users per 10,000 resident children aged 5–17 years) are listed in Table 1. The number of prevalent and new users who were dispensed antipsychotics increased over time among both girls (Fig. 1a) and boys (Fig. 1b).

In 2008, 2,527 children aged 5–17 years were dispensed at least one prescription of an antipsychotic drug (12.3/10,000 girls and 20.8/10,000 boys were prevalent users, Table 1). The corresponding number in 2021 was 5,942 children (36.6/10,000 girls and 37.3/10,000 boys). The observed higher rate of prevalent users of antipsychotics among boys compared with girls at the beginning of the study period had thus levelled out in 2021. Nearly all children (99%) were dispensed SGAs in 2021.

**Table 1:** Dispensations of antipsychotics in children aged 5–17 years living in 2008–2021

Calendar year	2008	2009	2010	2011	2012	2013	2014	2015	2016	2017	2018	2019	2020	2021
Number of resident children, N	1,386,192	1,371,281	1,356,194	1,350,535	1,351,695	1,370,917	1,397,956	1,435,674	1,472,602	1,515,348	1,548,706	1,577,422	1,594,232	1,608,544
Girls, N	674,762	667,659	660,228	656,817	656,743	665,931	678,661	696,721	714,242	733,936	750,706	765,457	773,364	780,488
Boys, N	711,430	703,622	695,966	693,718	694,952	704,986	719,295	738,953	758,360	781,412	798,000	811,965	820,868	828,056
Prevalent users ^a^ of antipsychotic drugs, N (N/10,000 adj^b^)	2,527 (16.7)	2,563 (17.4)	2,717 (19.1)	2,782 (20.2)	2,934 (21.8)	3,141 (23.4)	3,486 (25.7)	3,752 (27.0)	4,002 (28.0)	4,203 (28.4)	4,587 (30.2)	5,020 (32.2)	5,388 (34.0)	5,942 (36.9)
Girls, N (N/10,000 adj^b^)	948 (12.3)	976 (13.1)	963 (13.3)	962 (13.9)	1,007 (15.2)	1,102 (16.8)	1,272 (19.4)	1,409 (21.1)	1,559 (22.7)	1,644 (23.2)	1,841 (25.2)	2,117 (28.1)	2,394 (31.2)	2,853 (36.6)
Boys, N (N/10,000 adj^b^)	1,579 (20.8)	1,587 (21.5)	1,754 (24.5)	1,820 (26.1)	1,927 (28.0)	2,039 (29.5)	2,214 (31.6)	2,343 (32.6)	2,443 (33.0)	2,559 (33.3)	2,746 (34.9)	2,903 (36.1)	2,994 (36.6)	3,089 (37.3)
Mean age (years) girls, prevalent users	14.8	14.8	14.8	14.7	14.5	14.5	14.4	14.5	14.6	14.6	14.6	14.6	14.6	14.6
Mean age (years) boys, prevalent users	13.6	13.6	13.4	13.3	13.3	13.3	13.2	13.1	13.2	13.2	13.3	13.2	13.3	13.3
New users^c^ of antipsychotic drugs^a^, N (N/10,000 adj^b^)	1,186 (7.9)	1,101 (7.5)	1,220 (8.6)	1,203 (8.7)	1,273 (9.4)	1,393 (10.3)	1,570 (11.5)	1,637 (11.7)	1,756 (12.3)	1,856 (12.5)	2,135 (14.0)	2,327 (14.9)	2,510 (15.8)	2,819 (17.5)
Girls, N (N/10,000 adj^b^)	529 (6.9)	486 (6.5)	477 (6.6)	489 (7.1)	506 (7.7)	594 (9.1)	690 (10.5)	728 (10.9)	819 (11.9)	848 (12.0)	1,022 (14.0)	1,167 (15.5)	1,327 (17.3)	1,617 (20.7)
Boys, N (N/10,000 adj^b^)	657 (8.8)	615 (8.4)	743 (10.5)	714 (10.3)	767 (11.1)	799 (11.5)	880 (12.5)	909 (12.5)	937 (12.6)	1,008 (13.1)	1,113 (14.1)	1,160 (14.4)	1,183 (14.5)	1,202 (14.5)
Prevalent users ^a^ of SGAs, N (N/10,000 adj^b^)	2,330 (15.5)	2,400 (16.4)	2,564 (18.1)	2,662 (19.4)	2,824 (21.0)	3,036 (22.6)	3,392 (25.0)	3,646 (26.2)	3,910 (27.4)	4,107 (27.8)	4,486 (29.5)	4,917 (31.5)	5,299 (33.4)	5,860 (36.4)
Mean age (years) girls, new users	14.7	14.6	14.8	14.6	14.3	14.3	14.3	14.4	14.4	14.4	14.4	14.4	14.4	14.5
Mean age (years) boys, new users	13.0	13.0	12.8	12.7	12.6	12.7	12.5	12.3	12.6	12.5	12.7	12.6	12.8	12.7
Girls, N (N/10,000 adj^b^)	836 (10.9)	883 (11.9)	882 (12.3)	896 (13.0)	955 (14.4)	1,049 (16.0)	1,225 (18.6)	1,347 (20.1)	1,504 (21.9)	1,584 (22.3)	1,777 (24.3)	2,048 (27.2)	2,343 (30.5)	2,807 (36.0)
Boys, N (N/10,000 adj^b^)	1,494 (19.8)	1,517 (20.6)	1,682 (23.6)	1,766 (25.4)	1,869 (27.2)	1,987 (28.8)	2,167 (31.0)	2,299 (32.0)	2,406 (32.5)	2,523 (32.9)	2,709 (34.4)	2,869 (35.7)	2,956 (36.2)	3,053 (36.9)
Prevalent users ^a^ of lithium, N (N/10,000 adj^b^)	113 (0.7)	133 (0.8)	136 (0.9)	126 (0.9)	115 (0.8)	112 (0.8)	114 (0.8)	127 (0.9)	129 (0.9)	150 (1.0)	138 (0.9)	149 (1.0)	168 (1.1)	170 (1.1)
Girls, N (N/10,000 adj^b^)	68 (0.8)	81 (1.0)	78 (1.0)	66 (0.9)	55 (0.8)	52 (0.8)	54 (0.8)	77 (1.2)	75 (1.1)	91 (1.3)	74 (1.0)	91 (1.2)	111 (1.4)	106 (1.4)
Boys, N (N/10,000 adj^b^)	45 (0.5)	52 (0.7)	58 (0.7)	60 (0.8)	60 (0.8)	60 (0.9)	60 (0.9)	50 (0.7)	54 (0.7)	59 (0.8)	64 (0.8)	58 (0.7)	57 (0.7)	64 (0.8)

^a^Prevalent user: at least one drug dispensation during the calendar year

^b^N/10,000 adj: Number per 10,000 standardised by sex and age-distribution in 2021

^c^New user: a first dispensation during the calendar year after a drug free period of at least two years

The proportion of new users among children increased from 7.9 (6.9 girls and 8.8 boys) in 2008 to 17.5 (20.7 girls and 14.5 boys) per 10,000 in 2021 (Table 1). The observed lower number of new users among girls as compared with boys in 2008 shifted to a higher number starting in year 2019.

Lithium was dispensed to 113 children aged 5–17 years in 2008 and 170 children in 2021. More girls than boys were prevalent users of dispensed lithium during 11 of the 14 studied years (Table 1).

**Fig. 2:**
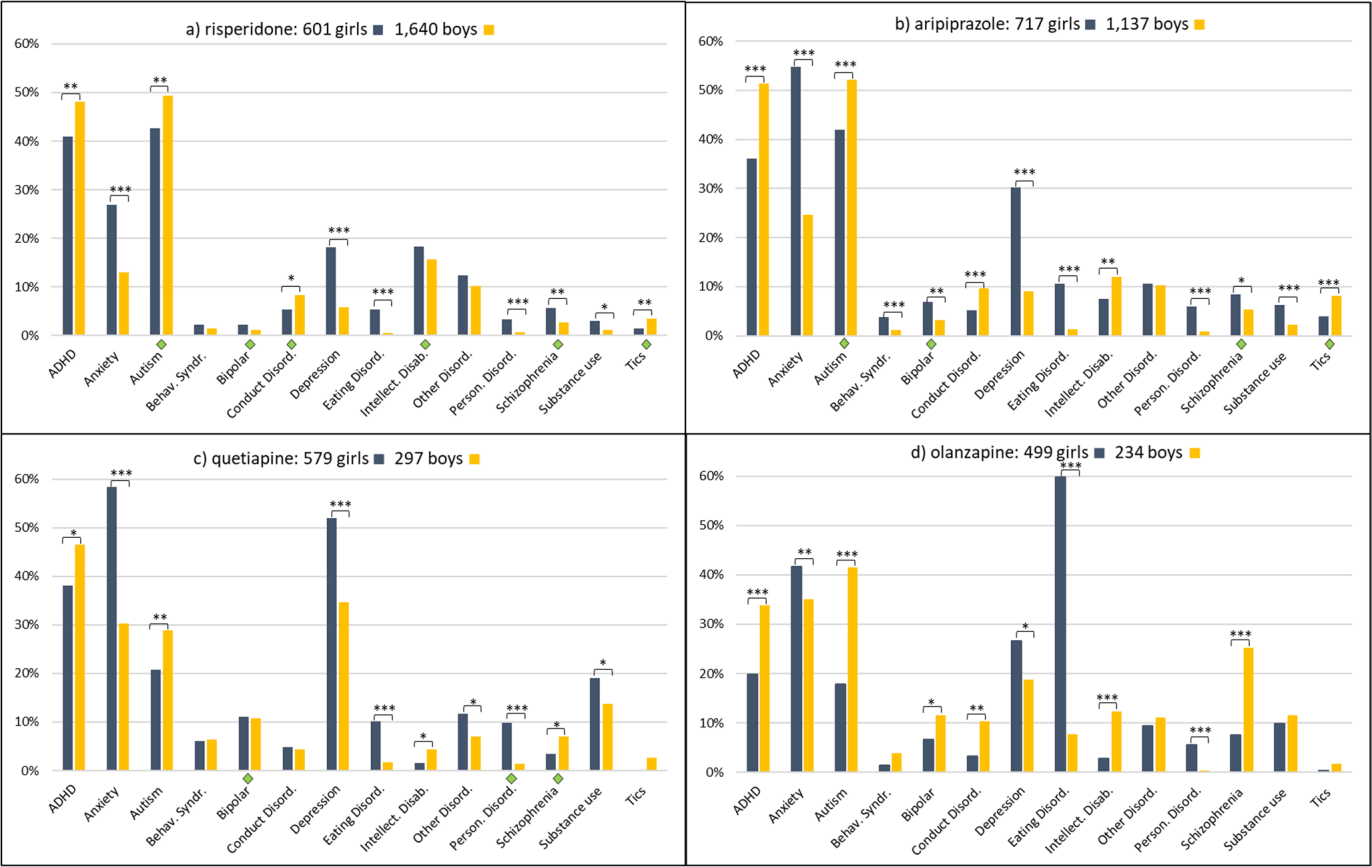
Relative frequencies of psychiatric disorders by sex in 2019 among children aged 5–17 years with at least one dispensation of risperidone (**a**), aripiprazole (**b**), quetiapine (**c**), or olanzapine (**d**). Green diamonds indicate on-label indications in accordance with the national guidelines from the Swedish Medical Products Agency and the Swedish Association for Child and Adolescent Psychiatry. Statistical significance between sexes indicated by *: *p* < 0.05; **: *p* < 0.01; ***: *p* < 0.001; no asterisk: *p* ≥ 0.05. No test for Tics (quetiapine and olanzapine) due to small samples. Each child can have more than one diagnosis registered

Focusing on the year 2019, 5,020 children aged 5–17 years had at least one dispensed prescription of an antipsychotic drug (2,117 girls and 2,903 boys). Table 1. This corresponds to a prevalence of 28.1/10,000 girls and 36.1/10,000 boys in 2019. The most dispensed antipsychotics in 2019 were risperidone (2,241 children), aripiprazole (1,854 children), quetiapine (876 children), and olanzapine (733 children), Fig. 2.

In 2019, most studied children (62% of girls and 69% of boys) across all age groups had three or more dispensed prescriptions of antipsychotics (i.e., persistent users). The highest frequency of three or more dispensations was observed for risperidone in children aged 12–17 years (girls 67% and boys 70%), Table 2. One dispensation only (during 2019) was most common for risperidone in girls aged 5–11 years (34%) and quetiapine in girls aged 12–17 years (34%), Table 2.

**Table 2:** Children aged 5–17 years by number of dispensations of antipsychotics, sex, and age group in 2019. The age group 5–11 years was omitted for olanzapine and quetiapine due to small numbers. Prescriptions to children using dispensable doses were excluded

	Age group			
	5–11 years, *N* (%)		12–17 years, *N* (%)	
Number of dispensations	Girls	Boys	Girls	Boys
Any antipsychotic				
All	**259 (100)**	**847 (100)**	**1**,**821 (100)**	**2**,**017 (100)**
1	76 (29)	193 (23)	431 (24)	341 (17)
2	49 (19)	119 (14)	250 (14)	249 (12)
3 or more	134 (52)	535 (63)	1140 (63)	1427 (71)
Risperidone				
All	166 (100)	640 (100)	433 (100)	986 (100)
1	56 (34)	168 (26)	97 (22)	173 (18)
2	26 (16)	104 (16)	45 (10)	123 (12)
3 or more	84 (51)	368 (58)	291 (67)	690 (70)
Aripiprazole				
All	93 (100)	281 (100)	620 (100)	847 (100)
1	26 (28)	80 (28)	160 (26)	179 (21)
2	22 (24)	43 (15)	94 (15)	115 (14)
3 or more	45 (48)	158 (56)	366 (59)	553 (65)
Quetiapine				
All			563 (100)	281 (100)
1			190 (34)	91 (32)
2			109 (19)	55 (20)
3 or more			264 (47)	135 (48)
Olanzapine				
All			480 (100)	203 (100)
1			138 (29)	58 (29)
2			76 (16)	31 (15)
3 or more			266 (55)	114 (56)

As regards the strengths of tablets dispensed in 2019, the proportions of children dispensed only the lowest strength were 21% for risperidone (0.25 mg) and 32% if oral solution 1 mg/ml was included; 86% for aripiprazole (5 mg) and 89% if oral solution 1 mg/ml was included; 48% for quetiapine (25 mg); and 50% for olanzapine (2.5 mg). Further, the proportions prescribed a maximum of one tablet of the lowest strength per day were 10% for risperidone, 69% for aripiprazole, 13% for quetiapine, and 25% for olanzapine.

Almost all children with dispensed antipsychotics had at least one psychiatric diagnosis in specialised in- or out-patient care (96%) in 2019, with an average of 1.8 to 2.4 diagnoses depending on substance. 42% of the children in 2019 had one single diagnosis registered. The type of psychiatric diagnoses among children with at least one dispensed antipsychotic drug varied substantially between different substances, Fig. 2a–d. For risperidone, autism and ADHD constituted the most registered diagnoses and were most common among boys (50% and 48%, respectively), Fig. 2a. For aripiprazole anxiety disorder and autism were the most common diagnoses among girls (55% and 43%, respectively) and autism and ADHD were most common among boys (53% and 51%, respectively), Fig. 2b. For quetiapine, anxiety disorders and depression were the most common diagnoses being especially common in girls (58% and 52%, respectively), Fig. 2c. For olanzapine, eating- and anxiety disorders were most common among girls (60% and 42%, respectively), and autism among boys (43%), Fig. 2d.

The different diagnoses showed similar prevalence rate across substances although the majority differed statistically significantly between the two sexes, Fig. 2a–d. Anxiety and depressive disorders were more common diagnoses in girls for all substances, whereas ADHD and autism were more common in boys. Two exceptions were noted. A diagnosis of schizophrenia was more common in girls receiving risperidone (Fig. 2a) or aripiprazole (Fig. 2b) than in boys on those medications. However, in children treated with quetiapine (Fig. 2c) or olanzapine (Fig. 2 d) a larger proportion of boys than girls were diagnosed with schizophrenia. For bipolar disorders, similar difference was observed: a larger proportion receiving aripiprazole were girls than boys whereas for olanzapine, the proportion of boys was larger than that of girls. No statistical differences between the sexes regarding bipolar disorders were observed for risperidone or quetiapine.

Among the children with dispensations of antipsychotics in 2019, the proportion prescribed within the paediatric labelling in the SmPC ranged from 0% to 28% for girls and from 0% to 25% for boys. When national guidelines from the Swedish Medical Products Agency and Swedish Society for Child and Adolescent Psychiatry were considered, the corresponding ranges of proportions were from 0% to 64% for girls and from 0% to 71% for boys, Table 3. The proportion of on-label use in accordance with national guidelines was highest for children dispensed risperidone, 71% in boys and 64% in girls, Table 3. For aripiprazole, the second most dispensed drug, the corresponding numbers were 65% in boys and 55% in girls. Quetiapine was 100% off-label according to the SmPC; using national guidelines the proportion on-label for quetiapine was 18% for boys and 23% for girls. Olanzapine was 100% off-label according to the SmPC and national guidelines. According to national guidelines, autism is the primary on-label diagnosis among children dispensed either risperidone or aripiprazole, Fig. 2a and b.

**Table 3:** Proportion of on-label prescriptions in the calendar year 2019 by sex among children aged 5–17 years with at least one dispensation of aripiprazole, risperidone, quetiapine, or olanzapine^a^

	Girls	Boys	Difference % (95% confidence interval)
Number of children with antipsychotics (*N*) and proportion on-label (%)			
Risperidone (*N*)	567	1,527	
On-label SmPC	34.7%	31.8%	2.9 (7.5, −1.7)
On-label national guidelines	64.2%	70.7%	−6.5 (−11.1, −2.0)
Aripiprazole (N)	**705**	**1**,**102**	
On-label SmPC	12.5%	7.0%	5.5 (2.6, −8.4)
On-label national guidelines	55.3%	65.2%	−9.8 (−14.5, −5.2)
Quetiapine (N)	**571**	**288**	
On-label SmPC	0	0	
On-label national guidelines	23.3%	17.7%	5.6 (−0.0, −11.2)
Olanzapine (N)	**497**	**225**	
On-label SmPC	0	0	
On-label national guidelines	0	0	

^**a**^On-label was classified both based on the paediatric labelling in the SmPC and the national guidelines from the Swedish Medical Products Agency and the Swedish Association for Child and Adolescent Psychiatry

Most prevalent users also had at least one dispensation of another psychotropic drug in 2019, among both girls and boys aged 5–17 years, Table 4. More than two-third of girls and boys were dispensed hypnotics and sedatives. Nearly two-thirds of girls were dispensed antidepressants and anxiolytics, and half of all boys were dispensed psychostimulants, Table 4.

**Table 4:** Dispensation of other psychotropic drugs in 2019 to children aged 5–17 years with at least one dispensation of antipsychotics, presented by sex

	*N*^a^ (proportion %)	
	Girls (*N* = 2,117)	Boys (*N* = 2,903)
Any psychotropic drugs	1,984 (94)	2,616 (90)
Hypnotics and sedatives (N05C)	1,405 (66)	1,856 (64)
Melatonin (N05CH01)	1,296 (61)	1,761 (61)
Other drugs (N05C excluding N05CH01)	401 (19)	305 (11)
Psychostimulants (N06B)	714 (34)	1,483 (51)
Anxiolytics (N05B, R06AD)	1,251 (59)	1,121 (39)
Antidepressants (N06A)	1,332 (63)	1,100 (38)
Antiepileptics (N03A)	237 (11)	286 (10)

^**a**^Each child can have more than one dispensed psychotropic drug

## Discussion

This nationwide register-based study revealed a substantial increase in dispensing of antipsychotics to paediatric patients in Sweden 2008–2021, among both girls and boys. The proportion of girls dispensed antipsychotics increased to equal that of boys. Girls had surpassed boys in new dispensations of antipsychotics at the end of the study period. Nearly all children were treated with SGAs, and most studied children were dispensed three or more prescriptions. Risperidone was most dispensed, followed by aripiprazole. The actual indications for prescribing were not registered in a structured manner; however, the patient register showed that girls treated with antipsychotics most often suffered from anxiety, depression, or eating disorders, whereas boys were most often diagnosed with ADHD or autism. Most children were dispensed the lowest available strength antipsychotics except in the case of risperidone, which was used also in higher doses. A majority of all dispensations were off-label according to the SmPC, but less markedly so when compared with national guidelines.

The findings of a marked increase in dispensed antipsychotics, in both girls and boys, was in line with other studies [8, 10, 14, 23]. A similar rate of drug prescriptions was observed in Norway, whereas Denmark showed slightly lower rates [10]. In our study, we found that the number of new users of dispensed antipsychotics among girls was substantially lower in 2008 compared with among boys, higher in 2021. A similar shift was observed in a Finnish study [23], though this has not been seen in other studies [10, 14]. The substantial reported increase in use of antipsychotics could be a result of the increased number of children and adolescents seeking care for mental health issues and receiving psychiatric diagnoses [4]. Furthermore, mental health related emergency department visits have increased among youths (6–24 years) [24]. Dinnissen et al. reported that antipsychotic prescriptions were not always combined with psychosocial treatment, which could indicate a lack of multimodal treatment in children and perhaps also suggests that antipsychotics are used somewhat too casually [25]. The mental health care utilisation pattern suggest that adults use psychological treatment to a lesser extent than pharmacological treatment [26]. Gangapersad et al. report a moderate increase in antipsychotic prescribing among adolescents during the COVID-19 pandemic, most pronounced in females aged 13–19 years [27]. In our data, this increase does not appear to follow the same trend in relation to COVID-19, a finding that is also supported by others [28]. 

The use of FGAs has declined in preference of SGAs, which are recommended for use in children [14, 29]. Risperidone is the most frequently prescribed antipsychotic drug across the Nordic countries as well as in other European countries [10, 14]. However, quetiapine was more commonly used in Denmark and Norway than in Sweden [10]. In Sweden, lithium use is higher than in other countries, although it remains uncommon. The increased use of lithium during the studied period was modest compared with the sharp increase in use of antipsychotics.

Psychiatric diagnoses among children treated with antipsychotics in our study varied substantially between different substances and between girls and boys. The observed differences between the two sexes were similar across substances and might reflect the underlying distribution of these conditions [4]. This finding was also consistent with the fact that many children were treated with lower doses, which may have other pharmacological effects on the psychiatric conditions that are more common in paediatric population. Especially quetiapine was to a large extent prescribed in 25 mg tablets, a considerably lower dose than what is common in antipsychotic treatment. Our findings regarding the use of low doses of antipsychotics are in line with results from a Scandinavian study [12]. The observed low dosing of quetiapine, combined with a diagnosis of depression, suggests that it may be used for other indications, such as sleep disorders. However, even low doses of antipsychotics can have severe metabolic side effects [7].

The finding that two thirds of all children treated with antipsychotics had off-label prescriptions according to the SmPC is consistent with findings from other studies [9, 25]. If instead considering recommendations in national guidelines, the level of on-label use of antipsychotics was higher. Still, at least one third of all children were treated off-label with antipsychotics. The observed diagnostic patterns indicates a substantial off-label drug use. Considering that many children seem to use antipsychotics for long periods (three or more dispensations in one year), a large number of children are at risk of suffering from clinically relevant and potentially serious side effects [30]. Some medications, such as olanzapine, were used in children even though this is discouraged due to the substantial risk of serious side effects. Most studies on paediatric efficacy and the safety of antipsychotic treatment are short-term and although these have shown treatment benefits in children, there is insufficient evidence on the effects of long-term antipsychotic use [3]. Meanwhile, there is clear evidence of antipsychotics uses being related to diabetes type 2 [31] and increased risk of metabolic syndrome in children and adolescents [32].

Nearly all children with at least one dispensed antipsychotic drug were also treated with at least one other psychotropic drug, in particular melatonin in both sexes, psychostimulants in boys, and antidepressants and anxiolytics in girls, which is consistent with results from other studies [13, 33, 34]. Increasing use of antipsychotics might be regarded as a result of the parallel trend to use drugs for other psychiatric disorders [8, 35]. Such concomitant psychotropic treatment may expose children to additional risks of side effects.

This use of antipsychotics, potentially for long durations and for indications outside both the approved labelling and national guidelines, exposes children to well-known safety risks, such as metabolic disorders, but also to possible unknown long-term risks. Intensified safety surveillance and further monitoring of use is needed to ensure safer and more rational paediatric use of antipsychotics.

## Strengths and limitations

A strength of this study was the coverage of all children in Sweden with dispensed antipsychotics during the study period, including unique individual data on diagnoses. Other advantages include the excellent data on dispensed drugs, which were likely to provide a more accurate picture of drug consumption than relying on prescriptions, as those may not always be dispensed [36].

A limitation was that information on indications was not included in the National Prescribed Drug Register and had to be deduced from contemporaneous diagnoses in the National Patient Register. The documented diagnoses are not always congruent with the clinically relevant diagnosis, and each child could have several diagnoses. We were not able to fully determine whether the increased utilisation of antipsychotics was driven primarily by new users, increased persistence among existing users, longer durations of use, or a combination of these factors. Antipsychotics given to hospitalised children were not included, as data on this were not possible to link to any specific patient using register data.

## Conclusions

Paediatric utilisation of antipsychotics has increased. The proportion of girls with dispensations has increased, equalling that of boys. More girls than boys were started on antipsychotics during the latter part of our study period up to 2021. The observed diagnostic patterns indicate considerable off-label use, raising questions regarding rational and safe use of antipsychotics in children – which should be addressed in future studies to identify signs of possible inappropriate drug utilisation in this population.

## Supplementary Information

Below is the link to the electronic supplementary material.

Supplementary Material 1 (DOCX 30.6 KB)

## Data Availability

The data are not publicly available due to Swedish secrecy laws. After approval from the Swedish Ethical Review Authority (https://etikprovningsmyndigheten.se), researchers can apply for data from the National Board of Health and Welfare, Stockholm, Sweden (https://www.socialstyrelsen.se).
