# Supplementary material for: Drug utilisation of antipsychotics and lithium in Sweden 2008–2021 – a nationwide study of children aged 5–17 years
Source: Eur Child Adolesc Psychiatry. 2026 Jan 10;35(5):1487–96. doi: 10.1007/s00787-026-02960-5 (PMC13272204; doi:10.1007/s00787-026-02960-5)
Supplement: Supplementary file 1 — Supplementary Material 1 (DOCX 30.6 KB) [file 787_2026_2960_MOESM1_ESM.docx]

**Supplementary**

**Table S1 Antipsychotics authorised by European Medicines Agency (EMA) presented by age, indication and dose**

| **Type of substance** | **Age** | **Indication** | **Daily dose mg in SmPC^a^ (weight of child)** |
| --- | --- | --- | --- |
| **Aripiprazole** | 13 years | Manic episodes in bipolar disorder | **10 mg** |
|  | 15 years | Schizophrenia | **10 mg** |
| **Lurasidone** | 13 years | Schizophrenia | **37 mg** |
| **Risperidone** | 5 years | Conduct disorders in children with intellectual disability | **0.5 mg (< 50 kg)**  **1 mg (≥ 50 kg)** |
| **Ziprasidone** | 10 years | Manic episodes in bipolar disorder | **120-160 mg ( ≥ 45 kg)**  **60-80 mg (< 45 kg)** |

**^a^**Summary of Product Characteristics

**Table S2 Categorisation of Psychiatric diagnoses**

| **Psychiatric diagnoses** | **ICD-10 SE codes** |
| --- | --- |
| Anxiety Disorders | F4 |
| Attention Deficit Hyperactivity Disorders (ADHD) | F90 |
| Autism Spectrum Disorder | F84 |
| Behavioral Syndromes | F51, F52, F53, F54, F55, F59 |
| Bipolar Disorders | F30, F31 |
| Conduct Disorders | F91 |
| Depressive Disorders | F32, F33, F34, F38, F39 |
| Eating Disorders | F50 |
| Intellectual Disabilities | F7 |
| Other Disorders | F92, F93, F94, F98, F99, F80, F81, F82, F83, F88, F89 |
| Personality Disorders | F6 |
| Schizophrenia | F2 |
| Substance use Disorders | F1 |
| Tic Disorders | F95 |

**Table S3. Categorisation of diagnosis based on on-label according to Summary of Product Characteristics (SmPC) and national guidelines (NG)^a^**

| **Psychiatric diagnosis** | **Risperidone** | | **Aripiprazole** | | **Quetiapine** | | **Olanzapine** | |
| --- | --- | --- | --- | --- | --- | --- | --- | --- |
|  | **SmPC** | **NG** | **SmPC** | **NG** | **SmPC** | **NG** | **SmPC** | **NG** |
| Anxiety Disorders | NO | NO | NO | NO | NO | NO | NO | NO |
| Attention Deficit Hyperactivity Disorders (ADHD) | NO | NO | NO | NO | NO | NO | NO | NO |
| Autism Spectrum Disorder | NO | YES | NO | YES | NO | NO | NO | NO |
| Behavioral Syndromes | NO | NO | NO | NO | NO | NO | NO | NO |
| Bipolar Disorder | NO | YES | YES | YES | NO | YES | NO | NO |
| Conduct Disorders | YES | YES | NO | NO | NO | NO | NO | NO |
| Depressive Disorders | NO | NO | NO | NO | NO | NO | NO | NO |
| Eating Disorders | NO | NO | NO | NO | NO | NO | NO | NO |
| Intellectual Disabilities | YES | YES | NO | NO | NO | NO | NO | NO |
| Other Disorders | YES | NO | NO | NO | NO | NO | NO | NO |
| Personality Disorders | NO | NO | NO | NO | NO | YES | NO | NO |
| Schizophrenia | NO | YES | YES | YES | NO | YES | NO | NO |
| Substance use Disorders | NO | NO | NO | NO | NO | NO | NO | NO |
| Tic Disorders | NO | YES | NO | YES | NO | NO | NO | NO |

^a^National guidelines published from the Swedish Medical Products Agency and the Swedish Society for Child and Adolescent Psychiatry.
